# A Comprehensive Review of the Pharmacological Importance of Dietary Flavonoids as Hepatoprotective Agents

**DOI:** 10.1155/2023/4139117

**Published:** 2023-04-21

**Authors:** Gajender, Avijit Mazumder, Ashwani Sharma, Md. A. K. Azad

**Affiliations:** ^1^Noida Institute of Engineering and Technology (Pharmacy Institute) 19, Knowledge Park-II, Greater Noida 201306, Uttar Pradesh, India; ^2^School of Pharmaceutical Sciences, MVN University, Palwal 121105, Haryana, India; ^3^Department of Pharmacy, Daffodil International University, Daffodil Smart City, Birulia 1216, Bangladesh

## Abstract

The liver is a crucial organ that is involved in various kinds of metabolic activity and a very stable accessory gland for the digestive system. Long-term or persistent inflammation and oxidative stress due to any reasons have a substantial impact on the beginning and continuation of chronic diseases such as hepatocellular carcinoma, liver cirrhosis, liver fibrosis, and other hepatic conditions. There are many sources which can help the liver to be healthy and enhance its metabolic potential of the liver. Since the diet is rich origin of bioactive along with antioxidant chemicals including flavonoids and polyphenols, it can control different stages of inflammation and hepatic diseases. Numerous food sources, notably vegetables, nuts, fruits, cereals, beverages, and herbal medicinal plants, are rich in bioactive chemicals called flavonoids and their derivatives like Flavones, Anthocyanins, Iso-flavonoid, Flavanones, Flavanols, and Flavan-3-ols. Most recently occurred research on flavonoids has demonstrated that they can regulate hepatoprotective properties. This is because they are essential parts of pharmaceutical and nutraceutical products due to their hepatoprotective, antioxidative, and immune-modulating characteristics. However, the characteristics of their hepatoprotective impact remain unclear. The purpose of this comprehensive review is to survey the flavonoid structure and enriched sources for their hepatoprotective and antioxidant effects concerning liver toxicity or injury.

## 1. Introduction

The liver is a crucial organ that is involved in various kinds of metabolic activity. Drug abuse, viral infection, excessive intake of alcohol, and biological and chemical agents, as well as correspondingly developed autoimmune assault of the hepatocyte, are just a few of the many reasons that can easily result in liver damage. Hepatic shape and function will be shattered and hampered if the damage cannot be reversed. Long-term hepatic damage could be a consequence of hepatic cirrhosis, fibrosis, fatty liver, and even also liver carcinoma, creating an epidemiological issue [1]. Currently, alternative treatment methods for several ailments are being embraced that use phytochemicals derived from natural resources. The lack of suitable and effective hepatoprotective drugs has remained a persistent worry despite significant advances in modern medicine. Due to the reason of drug-induced liver injury (DILI), many drugs have been taken off the market. Severe cases of liver failure can necessitate transplantation of the liver and result in organ death. Hepatic illnesses are primarily treated with plant-based medicines, and modern pharmaceuticals offer little alleviation for hepatic ailments. But there are not many medications on the market right now to address liver diseases. Due to their strength, purity, and affordability, herbal medications have gained importance and acceptance in recent years [2]. There are a huge number of bioactive substances, also known as secondary metabolites, in different plants, including alkaloids, polysaccharides, phenylpropanoids, Flavonoids, resins, tannins, steroids, essential oils, organic acids, anthraquinones, polyphenols, and saponins. This is because coevolution has produced so many of these substances [3]. More than five thousand polyphenolic chemicals with a fifteen-carbon core make up the vast family of flavonoids. Except for chalcones, which have a cleaved C ring, they frequently contain two phenyl rings A and B, and a heterocyclic C ring with attached oxygen [4]. The name of flavonoids comes from the Latin word known as flavus, whose meaning is “yellow,” which protects plants against oxidative damage in a defensive manner, male fertility, and visual signals [5]. Flavonoids are additionally known for their name as vitamin P for humans, it is a disputed name as they do not match the characterization of vitamins, and their influence on a variety of inflammatory diseases attracts research interest [5, 6]. Since the beginning of civilization, plants have been employed as traditional sources of therapeutic substances. The phytoconstituents derived from plants and their synthetic and semisynthetic analogues have been a major pathway to new pharmaceutical products since the advent of modern medicine and single pure medications [7]. Therefore, to effectively combat this threat, safe and affordable treatment alternatives must be developed. Natural secondary metabolites have been already taken on purpose for many centuries to treat various illnesses or diseases. Between 1981 and 2010, the FDA authorized about 35% of the medicines and their compounds that were obtained from natural sources. These natural substances already possess strong medicinal and anti-inflammatory properties. Numerous bioactive substances produced from plants, primarily flavonoids, can decrease inflammation by lowering the extent of potential mediators involving prostaglandin, Reactive oxygen species, and cyclo-oxygenase-2 as well as several cytokines like interleukin-6, interleukin-1, and TNF-*α*. Flavonoids are favourable to utilize in many dietary approaches due to the wide range of bioactivities they have been linked to in the human body, including antimutagenic, antioxidant, antiviral, and anti-inflammatory effects [8].

## 2. Chemical Structure of Flavonoids and Their Subclasses

Plants are a major origin of flavonoids, which are phenolic metabolites that exist naturally. An orange extract that was assumed to be a novel class of vitamins during the 1930s was eventually identified as a flavonoid. Over 8000 distinct flavonoids have been identified so far and have been reported in the literature. Different plant tissues contain flavonoids either internally or externally [8]. The pharmaceutical and biological ascribe to flavonoids such as antimalarial, cytotoxic, anticancer, cardioprotective, hepatoprotective, antileishmanial, antitrypanosomal, anti-inflammatory, and neuroprotective antiamoebic and also effective in the therapy of age-related factors, diabetes, and Alzheimer's disease [8, 9]. These qualities are typically attributed to their capacity for metal chelation, activity in scavenging free radicals, and degree of specificity in protein binding ability. The basic ingredient of flavonoids, which come in a variety of shapes, is a fifteen-carbon skeleton linked to two benzene rings A and B by a C (heterocyclic pyrene) ring. Distinct types of flavonoids vary according to the degree of oxidation and arrangement of C-ring substitution, although each compound within a class differs in the sequence of A and B-ring substitution [8]. Flavonoids can be subdivided into numerous subgroups based on the carbon in the C ring that the B ring is attached to, as well as the degree of oxidation and substitution of the ring C. Flavonoids classified as isoflavones have a B ring joined to the 3^rd^ position of the ring C. Neo-flavonoids include those in which the B ring is attached in 4^th^ position, subgroups can be formed based on the structural characteristics of the ring C for all those in which ring B is attached at position 2^nd^. Flavonoids are subcategorized into different classes including flavanols or catechins, flavanols, anthocyanins, flavonols, flavones, flavanones, and chalcones which are given in Table 1 with their chemical structure [10]. Flavonoids naturally occur in plants in the form of glycosylated or methylated compounds because these structures have higher bioactivity, bioavailability, and stability. A biological tool called glycosyltransferase has been used to glycolyze flavonoids, catalyzing the conjoined of a sugar moiety to an aglycone part to produce glycosides. Similar to how methyltransferase attaches methyl moieties to aglycone to create methoxides, the hydroxyl group in flavonoids is methylated in the existence of this enzyme. Methylation can result in the formation of C-methylated or O-methylated molecules via the carbon or oxygen atom, respectively. The pharmacological and biological characteristics of the methylated product relative to its parent compound were dramatically altered as a result of the methylation of flavonoids, according to experimental data [11].

## 3. Flavonoids and Their Subclasses with Their Dietary Sources

Flavonoids, also known as dietary flavonoids, are a class of organic compounds with a variety of phenolic metabolites that are primarily present in beverages and plant-derived food, such as flowers, bark, cereals, herbs, fruits, cocoa, tea, and wine. Depending on their chemical structure, flavonoids are subcategorized into twelve distinct groups, and hardly six of them are relevant to the diet which is given in Table 2 [10].

### 3.1. Flavonols

The flavonols are one of the subclasses of flavonoids and are distinguished from flavones by having an oxo group at the 4^th^ position and a 2,3-double bond present on ring C, which permits conjugation between rings (A and B) and significantly changes redox characteristics of flavonols. Although, they are unlike flavones in that they possess a hydroxyl group at position three, the sole nonphenolic one. Even though flavonols and flavones are highly alike, neither reduction nor oxidation of the former into the former appears to happen in plants. Higher plants have a consistent distribution of flavonols that is widely dispersed in leaves, stems, fruits, and flowers. Some of the most extensively investigated aglycons comprise isorhamnetin, kaempferol, galangin, isorhamnetin, rhamnetin, quercetin, fisetin, and myricetin [12]. Onions, broccoli, tea, and fruit all contain flavonols, which are represented by the glycoside(s) quercetin and kaempferol [13]. Flavonols such as isorhamnetin quercetin and rhamnocitrin have hepatoprotective activity. Dietary flavonols are bioavailable compounds with significant therapeutic benefits such as free radical scavenging activity, hepatoprotective activity, cardioprotective, antiviral, antibacterial, and antineoplastic activity, and the metabolization operation also results in compounds with astonishing bioactivities as well as the equivalent precursors. The incorporation of these compounds in the individual diet is strongly advised because of their undeniable health-promoting qualities since they make excellent nutraceuticals and functional food ingredients. According to studies conducted in-vitro and in vivo, flavonols alter the enzymatic potency of several molecular targets, including kinases, phospholipases, ATPases, lipoxygenases (LOX), cyclooxygenases (COX), and phosphodiesterases, which can be found in the three primary types of colon, breast, and liver cancer. This has an impact on the corresponding biochemical pathways. There is a wide spectrum of antineoplastic properties in flavonols: they control the cell cycle and reactive oxygen species (ROS)-scavenging enzyme activities, promote apoptosis, and autophagy and reduce the multiplication and encroaching of cancer cells [12, 14].

### 3.2. Flavones

Flavones and flavonols share a similar structural makeup, although the OH (hydroxyl) group on the pyran ring C3 of flavones is less [15]. Different biological actions, such as cardioprotective, anti-inflammatory, antitumour, antioxidant, antiallergic, and hepatoprotective activity are demonstrated by flavones and their synthetic derivatives [16, 17]. Lutein and apigenin are the two flavones that are most prevalent in plant food. In addition, they are abundant in celery, artichokes, parsley, grains, and celery. Various mono- and di-glycosides of apigenin, as well as orientin and iso-orientin, are also found in black and green tea [18]. The electronic and molecular structures of parent flavone and polymethoxylated flavones were explored in the theoretical investigation. A chosen class of flavonoids comprises substances found naturally in synthetic flavone derivatives and citrus trees. They have a diversity of bioactivities both in vivo and in vitro, and they can also shield plants from ultraviolet radiation [19].

### 3.3. Iso-Flavonoids

This class of flavonoids is regarded as a group possessing a ring B linked at position C3 rather than C2 in the fundamental structure [20]. Although they only have a modest range among plants, they are primarily found in the leguminous family. Globally, the prime dietary origin of isoflavones is mung bean, common bean, and soybean [21]. Black peas and green beans both contain certain isoflavones, including daidzein, genistein, and glycitein, in very small amounts. There have also been reports of several isoflavonoid subtypes in microorganisms [22]. In addition, isoflavonoids can be altered through polymerization, hydroxylation, or methylation to form different isoflavonoids such as isoflavonols and isoflavones [23]. Isoflavonoids also have great promise for treating many disorders. Daidzein and genistein are commonly known as phytoestrogens because of their oestrogenic activity in various models of animals. They have antidiabetic, antioxidant, and also effective against *Leishmania infantum*, cytotoxicity against Hep G2 cell lines, and Leishmania amazonensis [24, 25]. Isoflavonoids are essential for preventing cataract development in diabetic and hypertensive animal models [26].

### 3.4. Flavanones and Chalcones

All citrus fruits, including lemons, oranges, and grapefruit, often include flavanones, another significant class of compounds, and examples of this group of flavonoids include naringenin, eriodictyol, and hesperetin. They are also termed dihydro flavones, having a saturated C ring; the double bond between positions 2^nd^ and 3^rd^ is saturated in the two subgroups of flavonoids, unlike flavones, which is the primary structural difference between them. The quantity of flavanones has dramatically increased over the previous 15 years. While the biosynthesis process for flavonoids synthesized the secondary metabolite chalcones and its derivatives. Its chemical name is 1,3-diaryl-2-propen-1-one and consists of 2 aromatic rings linked by 3C atoms. Majorly chalcones found in nature are polyhydroxylated aromatic by nature [27]. The potential of flavanones to neutralize free radicals has been related to numerous health advantages. Citrus fruits have a bitter flavour in both their juice and peel because of these substances. As a hypo-lipidemic, antioxidant, cholesterol-lowering, and anti-inflammatory agent, citrus flavonoids have noteworthy pharmacological effects [10]. Psoriasis can be treated with flavanones, and flavonoids reduce skin inflammation. According to the previous study, flavonoids having a flavanone backbone were helpful for skin penetration [28].

### 3.5. Flavan-3-Ols, Flavanols, or Catechins

The supreme intricate group of flavonoids is known as flavan-3-ols or flavanols. They do not possess a double bond on the 2^nd^ and 3^rd^ carbon, unlike other flavonoids. They consist of simple monomers, including catechin and also its isomer epicatechin, as well as oligomers (which range from dimers to decamers), polymers (more than 10 parts), and other derived compounds including thearubigins and theaflavins). Proanthocyanidins or condensed tannins are other names for the polymers and oligomers of flavan-3-ols. The monomeric flavan-3-ols' two chiral centres at 2^nd^ and 3^rd^ Carbon form 4 isomers for one and all degrees of B-ring hydroxylation, two of which, (1)-epicatechin and (2)-catechin, are widely distributed in the environment. Another isomer, including (2)-epiafzelechin, has a more restricted distribution. By altering the cell's redox homeostasis and preventing NF-*κ*B activation, flavan-3-ols are effective against inflammatory disorders. Typically, they are believed to be beneficial ingredients in a range of drinks, supplemental, herbal medicines, and whole or processed foods. In addition, they have an impact on the food's astringency, bitterness, sourness, sweetness, colour formation, and sweetness [8, 29]. Proanthocyanidin, which are oligomers of flavan-3-ols, are thought to be good sources in malt and barley and are the predominant source of free radical scavenging potential [30]. When it refers to nuts, pecans, and hazelnuts are excellent sources of proanthocyanidins, whereas almonds, cashews, roasted peanuts, and pistachios are only modestly rich in the compound. Flavan-3-ols are also present in sufficient amounts in rosemary, mint, sage, dark chocolate, and dill [31].

### 3.6. Anthocyanins

Anthocyanins are phenolic group-related coloured, water-soluble pigments. Even though the O atom in ring C of the fundamental flavonoid structure of anthocyanin has a positive charge, it is still regarded as one of the flavonoids. It is sometimes referred to as the flavylium ion (2-phenylchromenylium). Anthocyanin exists as a glycoside, whereas anthocyanidin is an aglycone. Delphinidin, peonidin, malvidin, petunidin, pelargonidin, and cyanidin are the anthocyanidins that are most frequently found in herbs. The anthocyanins that give fruits and vegetables their blue, purple, and red hues are abundant in them. Currants, grapes, berries, and several tropical fruits have significant anthocyanin content. Edible vegetables with high anthocyanin concentrations include leafy vegetables with hues ranging from red to purple blue, roots, grains, and tubers. Cyanidin-3-glucoside, one of the anthocyanin pigments, is the main anthocyanin present in the majority of plants. The colourful anthocyanin pigments have historically been used as a natural food colouring. Temperature, pH, structure, and light all have an impact on the colour and stability of these pigments. When the pH is raised, anthocyanins turn blue instead of red. While possessing a positive charge on the O atom of the ring C of the fundamental structure of flavonoid, anthocyanin is still characterized as one of the flavonoids. It is also known as 2-phenylchromenylium ion. As a phytopharmaceutical, choleretic agent, appetite stimulant, and for the treatment of numerous other disorders, it has traditionally been used in numerous ways. Substantial pharmaceutical or nutraceutical compounds are these colourful pigments. The bioavailability of anthocyanin as a nutraceutical is essential for preserving health and preventing disease [32]. The scientific community has been interested in anthocyanins because of their anticancer, antimetastatic, and antioxidant potential [33]. According to twelve scientific studies, anthocyanin is capable of reducing apoptosis, cholinergic dysfunction, synaptotoxicity, cognitive deficits, tau hyperphosphorylation, neuronal extracellular calcium, oxidative stress, and dysfunction amyloidogenic pathway in various models of Alzheimer's [34].

## 4. Flavonoid as a Hepatoprotective Agent

Flavonoids act as hepatoprotective agents by targeting different mechanisms. The liver is particularly susceptible to attacks from reactive oxygen species, and it is also recognized as a vital regulator that contributes significantly to the development of liver illnesses with a high prevalence. Mostly liver diseases are caused by hazardous chemical exposure such as carbon tetrachloride, D-galactosamine, peroxidized oil, chlorinated hydrocarbon, aflatoxin, dimethylnitrosamine, thioacetamide, viral infections like Hepatitis AeE, autoimmune diseases, high doses of drug use (such as acetaminophen, and antibiotics), and excessive alcohol consumption are the main causes of hepatic diseases [14, 36]. The most prevalent chronic liver disease, known as nonalcoholic fatty liver disease (NAFLD), affects a significant section of the global population. NAFLD is characterized by the deposition of fat (more than 5%) in the hepatocytes in the absence of moderate alcohol consumption or additional factors of liver disease, such as autoimmune diseases, viral hepatitis, and drug-induced disorders. The most frequent causes of chronic hepatic disease are alcohol-associated liver disease and nonalcoholic fatty liver diseases. They both seem to have steatohepatitis, cirrhosis, fatty liver/steatosis, hepatocellular carcinoma, and fibrosis, but there are some variances as well. In contrast, ALD is more likely than NAFLD to have inflammatory cell infiltration, venous sclerosis, and venous or intravenous fibrosis. Nonalcoholic fatty liver or NAFL (simple steatosis) and nonalcoholic steatohepatitis (NASH) are on the NAFLD spectrum. NASH can proceed to hepatocellular cancer and cirrhosis, both of which are diseases of the liver and are marked by hepatocyte enlargement, inflammation, and different rate of fibrosis. Although the exact aetiology of NAFLD is unknown, new research has indicated that oxidative damage brought on by hepatic steatosis and insulin resistance could be substantial contributors to the condition and may be crucial in the development of NASH. A discrepancy between the frequency of triglyceride inflow and the degree of clearance leads to fat build-up in the liver. Enhanced lipolysis in peripheral tissues is the primary cause of the majority of free fatty acids (FFAs) accumulated as triglycerides. Hyperinsulinemia, dietary fat, and enhanced lipogenesis all contribute to increased lipolysis as a result of these factors. Lipid peroxidation, oxidative stress, mitochondrial dysfunction, and inflammation in hepatic tissues can arise from all of this. Due to metabolic abnormalities caused by fat deposition in the hepatic tissues, which also leads to increased mitochondrial reactive oxygen species generation and endoplasmic reticulum stress which cause the onset of inflammatory steatohepatitis. NASH is an inflammatory condition that primarily activates Kuffer cells (KCs) and astrocytes, which promotes the build-up of collagen and causes liver fibrosis [36]. The primary regulator in numerous experimental hepatic injury models, particularly CCl_4_-induced hepatitis, is iNOS, COX- 2, and TNF-*α* are the proinflammatory genes that are induced by an early increase in TNF-*α* levels [37]. The five flavonoids such as luteolin, Hesperetin, 3′,4′-dimethoxy hesperetin, chrysin, and apigenin help in improved superoxide dismutase, catalase, total antioxidant capacity, decreased nitric oxide synthase, nuclear factor erythroid-derived 2-related factor, and heme-oxygenase-1. They hindered the NF-*κ*B signalling pathway's phosphorylation of I*κ*B*α*, NF-*κ*B, and IKK, along with the blood serum elevation of proinflammatory cytokines and alanine and aspartate aminotransferase (ALT and AST). In addition, five flavonoids reduced caspase family protein expression and raised the Bcl-2/Bax ratio to prevent hepatocyte apoptosis. These flavonoids appear to protect the liver from acute hepatic injury brought on by D-GalN/LPS [38].

### 4.1. In Vivo and In Vitro Studies

Numerous studies have shown that some flavonoids are having anti-inflammatory, antihistamines, hepatoprotective, and antioxidant activity. Kaemferol, quercetin, myricitrin, malvidin, luteolin, apigenin, epicatechin, and hesperetin were discovered to be efficient against liver damage. In addition, all have been shown to control the Nrf2 signalling pathway, suppress NLRP3 inflammasome, and inhibit autophagy, oxidative effects, and apoptosis, by decreasing malondialdehyde and nitric oxide. They also improve catalase, total antioxidant capacity, heme-oxygenase-1, superoxide dismutase, and nuclear factor erythroid-derived 2-related factor [38–41].

#### 4.1.1. Quercetin

There are many red, green, and purple-hued fruits and vegetables that contain quercetin, including red onion, green leafy vegetables, kidney beans, apples, red grapes, evergreen tea, and apple cider vinegar. However, quercetin is the compound that is most frequently studied [42, 43]. The biological activity of quercetin, which includes, antioxidant, antiviral, and anti-inflammatory properties, has been demonstrated to have a wide range of biological applications, involving liver protection. It can link with transition metal ions and neutralize free radicals. Glutathione production within the body may be influenced by quercetin, which is involved in the process by which SOD converts oxygen into H_2_O_2_ and subsequently catalysis the breakdown of H_2_O_2_ into H_2_O. GSH-Px (glutathione peroxidase) can precisely catalyze the reaction between GSH and H_2_O_2_, minimizing the production of peroxides and preserving regular physiological processes [44, 45]. Quercetin can be effective in cholestatic liver injury. Platelets in cholestatic hepatic diseases are depleted, lose platelet granules and are unable to perform their function. The pathophysiology of platelets hypofunction in the bile duct ligation model is influenced by the expression of the ORAI-1 gene. Quercetin enhances store-operated calcium entry mediated by ORAI-1 and may be a promising therapeutic target in hemostatic diseases [46]. Quercetin can be employed as a hepatoprotective drug since it can effectively prevent t-BHP-induced hepatic injury in mice. The preventive advantages towards acute hepatic injury could be attributed to the improved free radical neutralizing action, reduction in peroxidation of lipids, and enhanced antioxidant capacity [47]. Numerous advantages and therapeutic qualities of quercetin have been noted, including hepatoprotective efficacy against triptolide-induced hepatic injury. Before the administration of TP (Triptolide), treatment with Quercetin (20000, 50000, and 80000 *μ*g/kg) reversed the changes caused by TP in a dosage range, demonstrating the ability of quercetin to prevent Triptolide-induced liver damage. One pathway driving this response was the balance between Th17 and Treg cells shifting from Th17 dominance to Treg dominance. The retinoid-related orphan receptor-t, proinflammatory cytokines interleukin-6 and interleukin-17 as well as the Th17 transcription factor, which was controlled by the Tim-3, and TLR4-MyD88-NF-*κ*B signalling pathway, have also been substantially downregulated by quercetin [48]. There are various quercetin derivatives with hepatoprotective effects, for example, hyperoside. Hyperoside is a flavonol glycoside with yellow solids and quercetin as its aglycon is called hyperoside and also referred to as quercetin 3-O-*β*-D-galactopyranoside. It has the potential to operate antioxidant activity to prevent hepatic oxidative stress in liver diseases. The hydrogen peroxide-induced intracellular oxidative damage in HepG2 cell lines demonstrated the antioxidative effects of hyperoside [49]. Notably, in the presence of hyperoside, the modulation of hepatic mitogen-activated protein kinase, heme-oxygenase-1, nuclear factor erythroid 2-related factor 2, and may augment antiapoptosis and ultimately prevent chemically-driven hepatotoxicity in mice [50]. It was discovered that hyperoside, at a dose of 60000 *μ*g/kg, can ameliorate the liver from oxidative damage and control metabolites and enzymes related to glutathione by blocking CYP4502E1. In overview, hyperoside inhibits the growth of hepatic tumour cells, arrests the cell cycle, and significantly reduces the expression of QKI (quaking), the activity of the Yin Yang 1(YY1) complex, and the proliferation and metastasis of hepatic tumour cells [51].

#### 4.1.2. Hesperetin

The hesperidin metabolite hesperetin-7-glucoside is more bioavailable than hesperidin itself. Hesperetin (HSP) is a flavonoid that occurs naturally and has a variety of pharmacological properties. It is most frequently found in citrus fruits like grapefruit, lemons, tangerines, and sweet oranges. Hesperidin can be found in numerous fruits and vegetables, as well as other herbal preparations, besides lemons and sweet oranges. *Citrus aurantium, Citrus sinensis*, and *Citrus limon* are where it is most frequently found. On a wide scale, HSP has also been taken out of *Citrus aurantifolia* callus cultures. Mandarin and citrus fruit peels have high HSP concentrations [52–54]. 7-O-(2-(propylamino)-2-oxoethyl)-hesperetin, a hesperetin derivative (HD-4d) demonstrated anti-inflammatory and hepatoprotective effects on alcohol-induced hepatic injury in C57BL/6J mice, as well as observable anti-inflammatory effects in ethanol and lipopolysaccharide-induced RAW264.7 cells. In addition, we found that HD-4d downregulates the expression of inflammatory factors via increasing NLRP12 both in vivo and in vitro. Additional research revealed that HD-4d increased NLRP12, which in turn prevented the phosphorylation and activation of the p65 proteins. In conclusion, HD-4d stimulated NLRP12 through the NF-*κ*B pathway to lessen liver damage and inflammatory response [55]. In mice, acetaminophen (APAP)-induced acute liver damage was lessened by hesperetin. Hesperetin dramatically reduced the liver hepatocytes apoptosis caused by APAP, according to apoptosis analysis using terminal deoxynucleotidyl transferase dUTP Nick end labelling (TUNEL). Similarly, the activity of caspase 3 revealed that acetaminophen overdose considerably raised liver caspase 3 activity; however, dose-dependent hesperetin suppressed the raised in caspase 3 in the liver of mice subjected to a toxic dose of acetaminophen [56]. A monomer molecule produced from hesperetin called hesperetin derivative (HD-16) with a dose of (25000, 50000, and 100000 *μ*g/kg) showed hepatoprotective and anti-inflammatory effects on a variety of hepatic disorders. On mouse hepatic fibrosis brought on by carbon tetrachloride as well as on LX-2 cells (human immortalized HSCs) triggered by transforming growth factor beta 1 (TGF-*β*1), HD-16 demonstrated an antifibrotic effect both in-vivo and in-vitro. The AMPK/SIRT3 pathways were regulated by HD-16 to exert an antifibrotic effect. The extent of damage and inflammation in the fibrosis of mouse liver caused by CCl_4_ was reduced, according to pharmacodynamic studies, using HD-16 [57].

#### 4.1.3. Apigenin

Apigenin (APG) is a flavone belonging to the superfamily of flavonoids engaged with a keto group at the 4^th^ carbon and substituted by hydroxy groups at the 4′, 5^th^, and 7^th^ positions. It is a naturally existing polyphenol that is produced and retained in a diversity of fruits, vegetables, and sanitary plants including celery, vine spinach, chamomile, artichokes, propolis, oregano, and parsley, and it is found in the dried form of plants. The highest concentration of apigenin, 45.035 *µ*g/g, has been found in dried parsley. Additional sources of apigenin include celery seeds, which have 786.5 *µ*g/g, dried chamomile flowers, which have 300–5000 *µ*g/g, vine spinach, which has 622 *µ*g/g, and Chinese celery, which has 240.2 *µ*g/g [58, 59]. In recent hepatoprotective activity, apigenin was given at a dosage of 0.1-0.2 g/kg P.O for 1 week, which lessened the concentration of the enzyme ALT and AST and decreased the seriousness of hepatic failure. It is significant to mention that pretreatment of apigenin increased the liver's manifestation of the proteins PPAR*γ*, and Nrf-2 as well as the activities of catalase, glutathione reductase, superoxide dismutase, and glutathione S-transferase while decreasing the levels of hepatic NF-*κ*B. Apigenin was demonstrated to have a variety of biological effects, notably anti-inflammatory and antioxidant characteristics as well as a protective impact counter to D-galactosamine/lipopolysaccharide-induced hepatic lesion in mice. Its mode of action may be based on rises in Nrf-2-mediated antioxidant enzymes and modulation of NF-*κ*B/PPAR*γ*-mediated inflammation [40]. In addition, apigenin also ameliorates edifenphos (EDF)-induced liver toxicity by protecting hepatic cells and has a mitigating effect on mitochondrial deterioration. When EDF was taken orally, the intracellular antioxidant system had been disrupted, which led to the generation of intracellular reactive oxygen species. EDF, on the other hand, encourages harmful consequences such as DNA damage, oxidative stress, decreased potential of mitochondrial membrane, generation of reactive oxygen species, stimulation of caspase 3/9 action, and the development of histo-renal histopathological abnormalities. By directly scavenging free radicals or exerting antioxidant activity, APG reduced the apoptosis and oxidative damage caused by EDF. Apigenin is an efficient dietary antioxidant that ameliorates EDF-induced oxidative stress [60].

#### 4.1.4. Epicatechin

A monomeric flavanol termed epicatechin (EC) has five hydroxyl groups substituted at positions 3, 3′, 4′, 5, and 7. It is a polyphenol and catechin by nature. It is a secondary metabolite ubiquitously present in plants and fruits such as cocoa powder, chocolate, teas, and grapes in notable concentrations. Epicatechin is an antioxidant with hepatoprotective, anti-inflammatory, antiobesity, and anticancer effects. Through the suppression of apoptosis and inflammation, epicatechin seems to have a protective effect on the liver of mice with acute hepatic lesions brought on by APAP. By blocking TNF-*α*, interleukin-1b, interleukin-6, and additionally inflammatory reaction, EC reduced immunological response and pathological damage. EC also inhibited Bax and caspase 3, subsidence of the pathway of mitochondrial apoptosis, raised in Bcl-2 to improve its antiapoptotic activity and reduced hepatic damage [41]. A dosage of 0.02–0.04 g/kg of epicatechin also reduced the severity of the hepatic sinusoidal obstruction syndrome brought on by monocrotaline by declining liver inflammation and oxidative stress. Rats treated with EC had improved nuclear translocation of Nrf-2 and raised the output of its downstream antioxidant genes [61]. In hamsters, an amoebic liver abscess was successfully treated with epicatechin. 1.106 Entamoeba histolytica trophozoites were administered intraperitoneally to the Syrian golden hamster. The EC dose (10 mg/100 g, I.P.) reduced the advancement of the liver abscess by modifying the influence of the inflammatory cytokines including interleukin-10, interleukin-1, TNF-*α* and aids in the removal of trophozoites to repair the liver [62].

#### 4.1.5. Malvidin

A naturally occurring flavonoid substance included in plant foods is malvidin, one of the anthocyanins. An O-methylated anthocyanin derivative is a malvidin. It is a fundamental plant pigment, and its glycoside is very prevalent. Previous research showed that malvidin had a diversity of pharmacological properties, involving antioxidant, anticarcinogenic, and anti-inflammatory properties. Previous literature revealed that malvidin can be effective against LPS-induced acute hepatic damage in mice. Malvidin may be able to inhibit NLRP3 inflammasome activation, block proinflammatory cytokines, and mediators and reduce oxidative stress by up-regulation of the Nrf2 signalling cascade. It may also be able to attenuate autophagy and hepatic apoptosis. These are the main mechanisms by which malvidin may be able to reduce acute liver injury caused by LPS [39]. Malvidin-3-galactoside, often known as MV-3-gal, is the main anthocyanin monomer present in blueberries. This substance has a strong antioncogenesis effect on various organs, such as the liver. MV-3-gal was given to Huh-7 hepatocellular carcinoma (HCC) cell lines, which decreased cell division, stop cell cycle, and colony formation depending on the dose via inhibiting the MAPK, MMP, and PTEN/Akt pathways [63].

**Figure 1:**
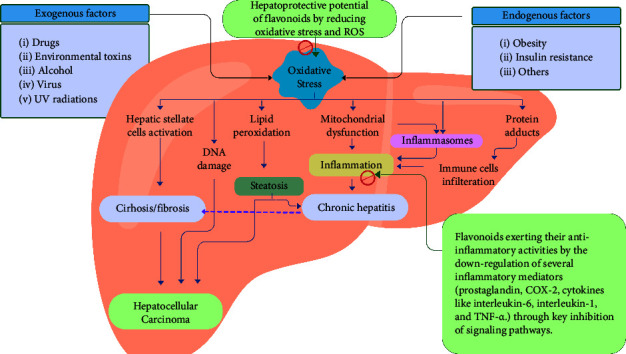
An illustration of induction of liver diseases by various factors via oxidative stress and inflammation along with the mechanism of action flavonoids as hepatoprotective agents, by inhibiting oxidative stress and inflammation.

Many exogenous and endogenous factors induce oxidative stress and cause hepatic diseases by various means from which oxidative stress and inflammation are highly concerned as they are the main targets of most flavonoids. For better understanding, an illustration of the induction of liver diseases by various factors via oxidative stress and inflammation along with the mechanism of action flavonoids as hepatoprotective agents, by inhibiting oxidative stress and inflammation is shown in Figure 1.

There are different mechanism of action of various flavonoids as a result of in vivo and in vitro research studies have shown in Table 3.

#### 4.1.6. Genistein

Genistein is an isoflavone that is primarily found in legumes such as fava beans, soybeans, kudzu, lupine, and Psoralea. The chemical name for genistein is 5,7-dihydroxy-3-(4-hydroxyphenyl) chromen-4-one [67]. A total of 15 carbons make up genistein, which is divided into two aromatic rings A and B, as well as one other carbon pyran ring (C), which contains the nucleus 3-phenylchromen-4-one. Positions two and three of the genistein basic carbon skeleton have a double bond. In addition, it has 3 more hydroxyl groups at positions 5^th^, and 7^th^ of ring A and the 4^th^ position of ring B and also has an oxo group at position 4 of ring C [68]. It was demonstrated to have displayed a broad range of pharmacological activities, such as anti-inflammatory, anticancer, antiproliferative, antioxidant, and protection against osteoporosis, and a reduction in cardiovascular-related diseases. It is an organic substance that resembles mammalian estrogens in terms of chemical structure. Numerous in-vivo and in-vitro research on the anti-inflammatory potential of genistein have been conducted in the past. The potent anti-inflammatory activity of genistein is achieved by blocking several signalling pathways, including those involving prostaglandins, nuclear factor kappa-B, proinflammatory cytokines, induced nitric oxide synthase, and reactive oxygen species [69]. In a rat model of chronic liver injury, hepatic damage, and hepatic fibrosis caused by D-GalN, the hepatoprotective effect of genistein was assessed at a dose of (250 mg/kg BW) two times a week for three months. Genistein (5 mg/kg BW) was administered intragastrically as a cotreatment once a day for 12 weeks, and the functional impairment that resulted was significant amelioration. This improvement included inhibition of activation of reduced expression of SMA (alpha-smooth actin) and deposition of the collagen matrix, as well as a rise in aspartate transaminase, serum alanine transaminase and also leads to elevated expression of hepatic Smad7 [64]. Another study found that supplementing alongside 150 mg/kg of genistein reduced the rise in ALT (alanine transaminase), AST (aspartate transaminase) activity, the decline in GSH (glutathione) level, and the amount of hepatic malondialdehyde in response to an inappropriate dose of acetaminophen [70]. Acetaminophen is a broadly used analgesic and antipyretic [71]. It is safe when taken at the appropriate dose, but an inappropriate dose of APAP (acetaminophen) can cause serious liver damage, which is one of the major factors for acute liver injury-related mortality [72]. At therapeutic concentrations, APAP is mostly metabolized by the enzymes of the UDP-glucuronosyltransferase 1A subfamily and sulfotransferase into glucuronide and sulfate conjugates, respectively. NAPQI (N-acetyl-p-benzoquinone imine), a lethal and extremely toxic metabolite is generated when a tiny amount is oxidized by the CYP family, particularly CYP2E1. By covalently joining with glutathione to generate a nonreactive metabolite, small levels of the metabolite are effectively detoxified and eliminated from the body by the liver. However, the glucuronidation and sulfation pathways saturate within 1 to 2 hours of an APAP overdose. Then, while drug consumption is too high, CYP2E1 produces NAPQI. Large amounts of NAPQI cause the liver's GSH to be rapidly depleted and covalently bind to mitochondrial proteins to produce reactive oxygen species, which in turn leads to hepatocellular necrosis and harsh centrilobular hepatotoxicity [73]. The sirtuins family includes SIRT1, which has been discovered as a NAD-dependent class 3 histone/protein deacetylase [74]. Widespread expression of SIRT1 and its role as a potent modulator on a variety of biochemical activities were becoming clearer, according to mounting data such as stress sensors [75], in metabolism [76], in cell survival [77], and autophagy [78].

#### 4.1.7. Daidzein

Daidzein is a compound with a low molecular weight that is synthesized from isoflavone, an isomer of flavone. Daidzein is a chemical compound that belongs to the class of 7-hydroxyisoflavones, which are 7-hydroxyisoflavones with an extended hydroxy group at position 4′. It is a dormant form of the tyrosine kinase inhibitor genistein. It is a phytoestrogen and a regulator of cellular proliferation, oxidative stress, and apoptosis. Daidzin is the equivalent glucoside form of daidzein, and through hydrolysis, daidzin can liberate daidzein. Daidzein is prevalent in many different types of vegetables, fruits, nuts, peas, lentils, seeds, and legumes, particularly in soybeans and foods and formulas based on soy. Epidemiology studies show an inverse relationship between cancer risk in the Asian population and a diet enriched in soy products [79–81]. Daidzein has reportedly been proven beneficial in treating LPS-induced hepatotoxicity. It acts by reducing oxidative stress and inflammation, hence diminishing the damage to the hepatocytes caused by LPS. Investigations were conducted into the mechanism underlying daidzein's impact on hepatocyte damage. Daidzein at a concentration of 100 *μ*M lowers AST and ALT expression levels without cytotoxicity. Moreover, 100 *μ*M daidzein reduced the expression of inflammatory markers such as interleukin-6, interleukin-1 beta, and tumour necrosis factor (58.8%, 73.8% 5.3%, and 55.5% 7.2%, respectively) in LPS-treated hepatocytes. In addition, 100 *μ*M daidzein boosted SOD activity by 88.418.9 and decreased LPS-induced ROS generation by 23.97.8% via upregulating Nrf2 expression and downregulating Keap-1 [65]. According to a different study, daidzein, chicory, and their combination significantly reduce the expression of the cyclin D1/CDK4 axis and the cyclin A/CDK2 axis in the liver tissue. This is accomplished by causing cell cycle arrest, preventing proliferation through the induction of apoptosis via the downregulation of Bcl-2, and suppressing the expression of Ki-67 [66].

## 5. Pharmacokinetics, Advantages, and Limitations of Flavonoids against Liver Diseases

In the case of liver damage, the pharmacokinetics of flavonoids may be altered due to changes in liver metabolism and clearance. For example, some studies have suggested that flavonoids may accumulate in the liver during liver disease, potentially leading to higher local concentrations and increased efficacy. On the other hand, other studies have reported decreased flavonoid bioavailability in patients with liver disease, which may limit their therapeutic potential. Flavonoids have shown countless health benefits, their low bioavailability has been a concern. Phase 2 metabolism is known to affect the bioavailability of flavonoids in humans. Usually, most flavonoids undergo sulfation, methylation, and glucuronidation in the small intestine and liver, and conjugated metabolites can be found in plasma after flavonoid ingestion. Despite the bioactivity expressed in different in vitro systems, the bioavailability of flavonoids would be a determinant factor of their bioactivity in vivo studies. Therefore, enhancement of bioavailability would be of utmost importance to exert health effects in, in vivo approach. In this regard, numerous attempts have been made to increase bioavailability such as improving intestinal absorption via the use of absorption enhancers, novel delivery systems, improving metabolic stability, and changing the site of absorption from the large intestine to the small intestine [82].

Flavonoids have been reported to have an excellent safety profile (no toxicity at up to 140 g/day), with no known significant adverse effects. The enthusiasm for flavonoids expressed by the public has sometimes overlooked their toxicity and also consumed the flavonoids exceeding the body's requirements. The consumption of a higher dose of flavonoids may lead to dysfunctioning in the multiorgan system. One of these approaches involves the use of kinetically stable nanoemulsion technology, where the lipophilic flavonoids can be prepared as emulsions consisting of extremely small particle sizes (<200 nm). The emulsified flavonoids are released slowly over time, allowing for a higher surface area for absorption, ultimately improving their absorption and bioavailability after oral administration. The advantages of using flavonoids in the treatment of liver diseases include their potential to reduce inflammation, oxidative stress, and fibrosis, all of which are common features of liver damage. In addition, flavonoids have been shown to have hepatoprotective effects, protecting liver cells from damage and promoting liver regeneration [83].

However, there are also limitations to the use of flavonoids in liver disease treatment. As mentioned, their low bioavailability can limit their effectiveness, and their metabolism in the liver may be altered in patients with liver damage. Flavonoids may also interact with other medications or supplements, potentially leading to adverse effects. Furthermore, the optimal dosing and duration of flavonoid supplementation for liver disease treatment are not well established, and more research is needed to determine their safety and efficacy in this context [84].

All the studies have revealed the flavonoids as hepatoprotective but some authentic information is required for their efficacy, dose, and potential for safe use of the flavonoids. For the same many clinical trials have been done and some are currently going on. The different flavonoids mentioned in this review were found to be effective against different liver-associated diseases and several studies of hepatoprotective activity of these in humans are clinically investigated an overview of important research on clinical trials of flavonoids for hepatoprotection is shown in Table 4.

## 6. Summary and Conclusion

Due to an increase in cases, liver disease or hepatotoxicity is a popular subject of growing concern. According to the above-given literature, liver conditions can be developed due to long-term inflammation. Due to antioxidant, hepatoprotective, and anti-inflammatory properties, dietary flavonoids are important in the development and mitigation of pathological conditions. Recent clinical studies of six major hepatoprotective flavonoids have confirmed beneficial effects on liver diseases by inhibiting inflammation by lowering thresholds of several cytokines including interleukin-1, interleukin-6, and TNF-*α*, decreasing its main mediator prostaglandins, COX-2, ROS, and preventing the NF-*κ*B/P65, IKK, and IKB*α* in the NF-*κ*B signalling. According to the research, flavonoids also prevent apoptosis of the liver by boosting the Bcl-2/Bax ratio and inhibiting the previously mentioned Caspase family protein. Hence flavonoids in diet could be a good option for preventing hepatic conditions. However, precise, dose-dependent clinical trials are still required in the future to evaluate the hepatoprotective potential of flavonoids to treat hepatic disorders with a safe approach.

## Figures and Tables

**Table 1:** Chemical structures of flavonoids and their major subclasses.

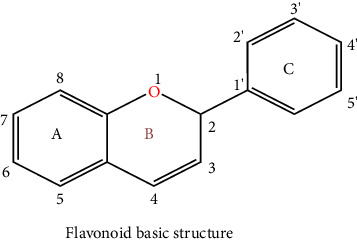	
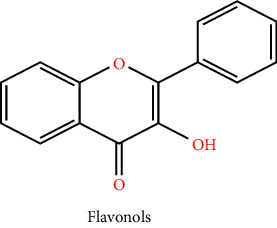	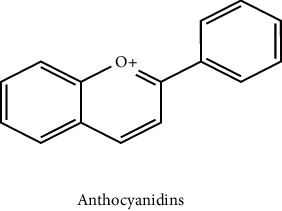
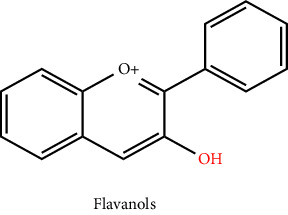	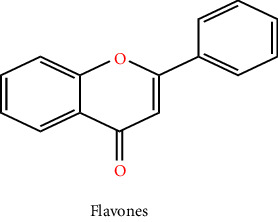
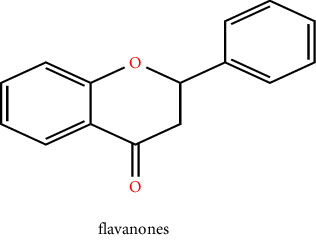	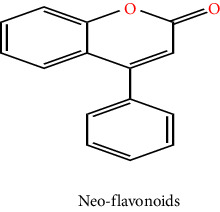
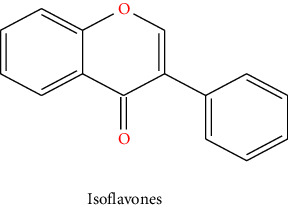	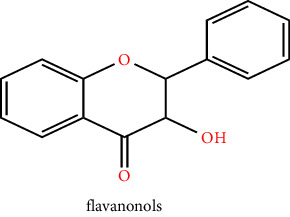

**Table 2:** Some hepatoprotective flavonoids are listed along with their various dietary origins.

Types of flavonoids	Subclasses	Mol. weight (g/mol)	Dietary origin	References
Flavones	Luteolin	286.24	Dried parsley, apples, peppers, oranges, watermelon, lettuce, iceberg	[35]
	Diosmetin	300.26		
	Baicalein	270.24		
	Apigenin	270.052		
	Chrysin	254.24		
	Ombuin	330.29		
Anthocyanins	Cyanidin	287.24	Blueberries, bananas, cherries, cabbage, cranberries, plums, beans, pears	
	Delphinidin	303.24		
	Pelargonidin	271.24		
	Peonidin	301.27		
	Petunidin	317.27		
	Malvidin	331.296		
Iso-flavonoid	Genistein	270.24	Strawberries, peaches, banana, tea, soya nuts, pears	
	Glycitein	284.26		
	Puerarin	416.4		
	Daidzein	254.23		
Flavanones	Eriodictyol	288.24	Grapefruit juice, orange juice, tomatoes, lemon juice, tangerines, tea	
	Hesperetin	302.27		
	Naringenin	272.257		
Flavonols	Silymarin	482.44	Tea, onion, tomatoes, apples, coffee, saffron	
	Fisetin	286.236		
	Myricitrin	464.37		
	Rutin	610.51		
	Kaempferol	286.23		
	Quercetin	302.23		
	Gossypin	318.23		
	Iso- rhamnetin	316.26		
	Galangin	270.24		
	Ayanin	344.31		
Flavanols, flavan-3-ols	Epicatechin	290.26	Apple, bananas, pears, strawberries, tea	
	Theaflavin	564.49		
	Thearubigins	902.7		

**Table 3:** Mechanism of action of various flavonoids as a result of in-vivo and in-vitro research studies.

Flavonoids	Liver injury	Mechanism of action	Conclusion	References
Quercetin	Cholestatic liver injury in rats	Increase ORAI-1 gene expression	Increased ORAI-1 gene-mediated SOCE leads to improved platelet function in the bile duct ligation model of cholestasis in rats	[46]
	t-BHP induced acute liver damage in mice	Scavenge free radical, increase in GSH, CAT, SOD, and decrease in serum enzyme markers i.e., ALT, AST	Through an increase in antioxidant activity, quercetin neutralizes free radicals and lowers oxidative stress	[47]

Hesperetin	Both ethanol and lipopolysaccharide-induced RAW264.7 cells and alcohol induce mouse liver	Enhance NLRP12 in two in vivo and in-vitro models which lowers inflammatory factors and prevents expression and phosphorylation of P65 proteins	Hesperetin stimulated NLRP12 through NF-*κ*B to lessen inflammation and liver damage	[55]

(HD-16) hesperetin derivative	Carbon tetrachloride-induced liver fibrosis and liver inflammation	*α*-SMA, Col3*α*1, TIMP-1, and Col1*α*1 expression were inhibited in TGF-1-stimulated LX-2 cells, and activating the SIRT3/AMPK pathway also reduced carbon tetrachloride-induced liver inflammation and fibrosis	Hesperetin derivative-16 attenuated carbon tetrachloride-induced liver fibrosis and inflammation	[57]

Apigenin	D- galactosamine/LPS-induced liver injury	Decrease in ALT, and AST, and raised the activity of superoxide dismutase, glutathione reductase, catalase, as well as Nrf-2 and PPAR*γ* protein expression, and decrease in hepatic TNF-*α*, and NF-*κ*B expression	Apigenin may ameliorate D-GalN/LPS-induced liver injury in mice by increased Nrf-2-mediated antioxidative enzyme and modulate the PPAR*γ*/NF-*κ*B mediated expression	[40]
	Edifenphos- induced toxicity by modulating ROS- mediated mitochondrial dysfunction, oxidative stress, and caspase signal pathway in rat liver and kidney	Apigenin ameliorated the edifenphos-induced oxidative damage via antioxidant activity or by scavenging free radicals	Apigenin reduces oxidative stress-induced damage in the liver	[60]

Epicatechin	Acetaminophen-induced acute hepatic injury of mice	Inhibit TNF-*α*, IL-6, and IL-1, reduce the pathway of mitochondrial apoptosis, increase Bcl-2 to amplify the antiapoptotic response, and inhibit bax caspase-3	In mice, epicatechin improves the antiapoptotic effect while decreasing the acute liver injury brought on by APAP	[41]
	Hepatic sinusoidal obstruction syndrome by inhibiting liver inflammatory and oxidative injury	In rats, epicatechin increased Nrf-2 translocation and the production of its downstream antioxidant gene	At a dosage of 0.02–0.04 g/kg, epicatechin reduced hepatic sinusoidal syndrome by reducing hepatic inflammation and oxidative stress	[61]

Malvidin	Lipopolysaccharide-induced acute liver injury	Reduce oxidative damage by activating the Nrf2 signalling cascade, reduce inflammation through blocking proinflammatory cytokines and mediators as well as NLRP3 inflammasome activity resulting attenuate apoptosis, and autophagy	Malvidin attenuated hepatocyte apoptosis and autophagy via an upregulating Nrf2 signalling pathway	[39]

Genistein	D-galactosamine induced liver fibrosis/chronic liver damage in rats	Inhibit activation of reduced expression in *α*-SMA and deposition of collagen matrix and elevation in blood serum AST, ALT and leads to increased expression of hepatocellular Smad7. Which finally muffles the expression of smad/TGF-*β*1 signalling	Alterations in histopathology brought on by D-GlN are substantially prevented by genistein (5 mg/kg BW)	[64]

Daidzein	LPS-induced hepatotoxicity	Inhibit inflammation and oxidative stress by reducing the level of AST, ALT, IL-6 IL-1*β*, and TNF and also increase the level of SOD by upregulating and downregulating Keap-1	Daidzein was found effective to ameliorate oxidative stress and inflammation in LPS-induced hepatotoxicity	[65]
	Hepatocellular carcinoma	Reduce the expression of the cyclin D1/CDK4 axis and the cyclin A/CDK2 axis in the liver tissue via downregulation of Bcl-2, and suppressing the expression of Ki-67	The daidzein and chicory combination was found to be effective to treat hepatocarcinogenesis	[66]

SODS = superoxide dismutases; SOCE = store opening calcium channel; TGF-*β*1 = transforming growth factor Beta 1; GSH = glutathione; ALT = alanine transaminase; CAT = catalase; AST = aspartate transaminase; NF-*κ*B = nuclear factor kappa B; SIRT3 = silent mating type information regulation 2 homolog 3; *α*-SMA = *α* smooth muscle actin; NLRP3 = nucleotide-binding domain(NOD)-like receptor protein 3; Bax = Bcl-2 associated *X* protein; Smad = mother decapentaplegic homolog; Nrf2 = nuclear factor erythroid 2-related factor2; APAP = acetaminophen; D-GlN = D-galactosamine; PPAR*γ* = peroxisome proliferator-activated receptor gamma; IL-6 = interleukin; IL-1*β* = interleukin-1 beta; LPS = lipopolysaccharide; t-BHP = tert-butyl hydroperoxide; TNF-*α* = tumor necrosis factor-alpha; ROS = reactive oxygen species.

**Table 4:** An overview of important research on clinical trials of flavonoids for hepatoprotection.

S.no	Clinical trial registration number	Phases/Study type	Study enrollment	Type of intervention/Treatment	Dose	Duration of trial	Endpoint/Outcome
1	NCT01438320	Phase 1	30 patients with chronic HCV	Dietary supplement of quercetin	Quercetin supplement (5 g daily)	2011-07 to 2014-06	No change in ALT, and AST levels. Quercetin exhibits a potential for antiviral activity in some hepatitis C patients
2	NCT05506488	Phase 1 Phase 2 Double-blind randomized controlled	30 patients	Drug: Dasatinib + quercetin Other: Placebo	Dasatinib (100 mg) + quercetin (1000 mg)	2023-03-01 to 2025--10	Due to the potential role of senescence in NAFLD-related fibrosis, dasatinib, and quercetin might thus be interesting future therapeutic options to tackle NAFLD-related fibrosis
3	NCT02309801	Phase 1/Interventional	10 patients (18 Years to 50 Years)	Dietary supplement: Daidzin Dietary supplement: alcohol	Alcohol (0.5 g/kg) and daidzin supplement (80 mg)	2012-07 to 2014-11	Daidzin exhibits disulfiram adverse effects and interferes with alcohol metabolism
4	NCT03278925	Phase 1	48 patients	Drug: Defined green tea catechin extract Other: Pharmacological study Other: Laboratory biomarker analysis Other: Questionnaire administration	Catechin extract orally (PO) once daily or twice daily (BID) for 24 weeks	2018-08-09 to 2023-03-31	Specified green tea catechin extract might be more effective at reducing *γ*-OHPdG levels and halting the growth of liver cancer
5	NCT01091077	Phase-1/Interventional and pilot study	7 patients (18 Years to 65 Years)	Dietary supplement: Naringenin Other: Placebo	1 gram of naringenin mixed with 16 grams of hydroxypropyl-*β*-cyclodextrin in 250 mL of water	2009-06 to 2013-08	The naringenin blocks the fat by which the virus is bound and lower the viral load in the bloodstream
6	NCT01940263	Early phase 1/Interventional a randomized, double-blinded, placebo-controlled trial	63 patients	Dietary supplement: Anthocyanin Dietary supplement: Placebo	Placebo 320 mg daily for twelve weeks Anthocyanin 320 mg daily for twelve weeks	2013-06 to 2014-06	In rodents with experimental NASH, anthocyanins from various plant sources have been demonstrated to reduce oxidative stress, dyslipidemia, liver steatosis, and inflammation
7	NCT01800487	Interventional and prospective, double-blind, placebo-controlled trial	80 patients	Drug: Silymarin Drug: Placebo	Silymarin (140 mg) thrice daily Placebo (140 mg thrice daily)	2012-01 to 2013-07	The study endpoints are the level of ALT by week 4 and the number of patients who developed antitubercular drug induced-DILI. Silymarin exhibits hepatoprotective activity by reducing the level of ALT, AST, and total bilirubin
8	NCT03749070	Interventional double-blind, controlled, randomized clinical trial	132 patients (20 Years to 60 Years (adult)	Dietary supplement: Silymarin	700 mg of silymarin	2019-02-15 to 2022-08-31	The endpoint of this study may provide a valuable opinion on whether silymarin can be used as adjuvant therapy for the management or treatment of NAFLD

## Data Availability

The supporting literature, data, and other necessary information used to support the findings of this study are available from the corresponding author upon request.
